# Patient-provider disconnect: A qualitative exploration of understanding and perceptions to care integration

**DOI:** 10.1371/journal.pone.0187372

**Published:** 2017-10-27

**Authors:** Yi Feng Lai, Andrew Yew Wai Lum, Emily Tse Lin Ho, Yee Wei Lim

**Affiliations:** 1 Department of Pharmacy, Sengkang Health, Singapore, Singapore; 2 Department of Pharmacy, Raffles Hospital, Singapore, Singapore; 3 Regional Health System, Singapore Health Services, Singapore, Singapore; 4 Saw Swee Hock School of Public Health, National University of Singapore, Singapore, Singapore; Hopitaux Universitaires de Geneve, SWITZERLAND

## Abstract

**Background:**

Integrated care has been well-recognized as a solution to improve quality of care for patients with complex needs. As Singapore increasingly develops and promotes integrated models of care, it is unclear if providers, patients, and caregivers share similar understanding of changes in the healthcare system.

**Objectives:**

This study aims at exploring three dimensions of care integration: a) understanding of integration; b) challenges and c) changes perceived as essential among three distinct stakeholder groups: providers, patients and caregivers.

**Methods:**

This qualitative study was conducted among 41 care providers (clinicians and administrators) and care consumers (patients and caregivers) in Singapore utilizing 29 semi-structured interviews and 2 focus group discussions. Study participants were selected by purposive, snowball sampling from various clinical settings. Data were transcribed, familiarized, coded and analyzed using a conceptual framework.

**Results:**

Understanding of care integration was generally lacking among patient and caregivers. Most of them focused on healthcare costs and accessibility of services. Providers characterized care integration in clinical process terms and had a more systems view of the concept. Most participants viewed resource constraints as a key challenge in integrating care. Additionally, providers expressed the need for patients and their families to play a greater role in managing their health. Individuals and the community are key components of an integrated care system in the future. Reliance on the healthcare system alone is not sustainable.

**Conclusions:**

Patients, caregivers and providers have varying degrees of understanding towards care integration. The success of engaging stakeholders on the ground to be active participants in the healthcare system integration process requires policymakers and healthcare leaders to increase patient engagement efforts and to better appreciate the challenges faced by the healthcare workers in the rapidly changing national and global healthcare landscape.

## Introduction

Integrated care is recognized to be a foundation for better quality of care and quality of life for patients with complex, long-term illnesses [1]. The World Health Organization (WHO) defined integrated care as “a coherent set of methods and models of funding, administrative, organizational, service delivery and clinical levels designed to create connectivity, alignment and collaboration within and between the cure and care sectors” [2, 3]. Care integration often involves connecting the acute and community healthcare systems with non-healthcare service systems [4–10]. In addition, care integration objectives are not just person-focused but also institution-focused and population-focused [11]. However, the interpretation of care integration in practice varies considerably. A review of the literature uncovered 175 interpretations of integrated care [12]. Apart from actual differences in scope of care integration, there were also instances of multiple phrases referring to a similar concept. For example, integrated care delivery mechanisms yielded terms such as “integrated delivery networks”, “integrated health networks”, and “integrated health delivery systems”.

Multiple interpretations of integrated care have contributed to heterogeneous understanding and operationalization of the concept in practice. From patients’ perspective, some may argue that care integration as a concept need not be fully understood. However, such lack of accurate understanding can result in misinterpretation of programs’ and policies’ intent by the public, which can then lead to unintended care utilization patterns and delivery challenges [13–15]. From clinicians’ perspective, differences in care processes and clinical workflow due to different care integration priorities and definitions can cause confusion and give rise to differences in expectation and what is achieved in reality in terms of quality of care [16–19].

Like many developed countries, Singapore is actively developing integrated healthcare systems. A recent initiative in the public healthcare sector was the formation of six Regional Health Systems (RHS). Each RHS was set up to encourage tertiary hospitals in a geographic region to work with healthcare providers such as step-down care facilities, nursing homes, general practitioners (GPs), and home care providers [20] to provide seamless care to patients along the care continuum. This was consistent with consensus and recommendations [21–23] of acute care providers taking leadership roles in their local health systems, and taking greater responsibility for disease prevention and health promotion. However, within a short span of a few years, Singapore saw another major restructuring process that halved the number of RHSes from six clusters to three [24–25]. Such rapid and drastic changes to the national healthcare landscape can potentially lead to confusions on the ground. In the primary care sector, there were also numerous programs that were launched nationwide in quick succession, including the Primary Care Partnership Scheme (PCPS), Community Health Assist Scheme (CHAS), Pioneer Generation (PG) package, Community Health Centers (CHCs) to encourage utilization of private primary healthcare. Consequently, it was unclear if the public actually understood the intent of these initiatives and if confusions arose as we develop new models of care in Singapore. Administrative barriers and lack of public awareness and understanding [26] could lead to failure of these programs. To date, there is no study investigating the understanding and its agreement to the care integration approach between various healthcare stakeholders in Singapore. These development and potential misinterpretation of policy intents motivated us to examine not only healthcare providers’ but also patients’ and caregivers’ understanding of the healthcare systems with the concept of care integration.

We attempted to answer three specific questions: 1) What do patients and providers understand about care integration? 2) What do patients and providers see as challenges in care integration? and 3) What essential changes in policy, practice, belief, and behavior do patients and providers expect to bring about successful integrated care?

## Method

### Context of the study

Singapore’s healthcare system is accessible through a wide network of primary, acute and step-down care providers. Patients are free to choose their providers, either in the government-subsidized public sector or private sector for all levels of care. Health insurance is not mandatory in Singapore and the healthcare financing model is primarily fee-for-service. Beyond out-of-pocket payment, Medisave (national medical savings scheme), Medishield (basic health insurance administered by the government) and Medifund (an endowment fund for needy patients) are the three pillars of its healthcare financing system to ensure no Singaporean is denied medical care due to financial reasons. The Ministry of Health and its statutory boards regulate both the public and private providers of healthcare in Singapore, and set standards and guidelines to ensure healthcare’s quality and affordability.

### Study sample and data collection

This qualitative study was conducted among 41 patients, caregivers and providers in two organizations- Singapore General Hospital, a Joint Commission International accredited Academic Medical Center, and Sengkang Health, a regional acute care hospital, in Singapore. Data collection was done through semi-structured interviews and focus group discussions (FGDs) between June and October 2015. Participant recruitment criteria included: (i) Care consumers: Patients and caregivers who have previously utilized healthcare services in Singapore, (ii) Care providers: healthcare professionals, administrators and leaders; (iii) Able to communicate in either English or Mandarin. Participants were excluded if they withdrew at any point of the data collection; or were unable or unwilling to give verbal consent for participation. The study protocol was approved by SingHealth Centralized Institutional Review Board (Ref: 2015–2317).

We selected healthcare workers and professionals whom we felt had relevant background at a strategic level, or were involved with intervention or programs related to care integration, such as work related to improving care coordination. Additional participants were identified by snowball sampling through recommendations from interviewed participants. For patients and caregivers, purposive convenient sampling was carried out in various care settings (inpatient and outpatient) and specialties (medical and surgical) until data saturation.

Areas covered in the interviews included: (1) Understanding of the concept of integrated care (e.g. What other terms does integrated care invoke? What are the aspects belonging to care integration? What does Regional Health System (RHS) mean to you?); (2) Problems and challenges perceived or encountered during care delivery or care seeking experience (e.g. Do you think it is easy for patients to navigate through the healthcare system? Do you think integrated care is a good idea to pursue?); and (3) Future needs and solutions going forward (e.g. If you may bring about change without any constraint, what will you change? How do you think care integration can affect the various stakeholders in the system?). During the conduct of the interviews, official definition of care integration was not given to the participants as this might influence the response elicited. Instead, if they were unable to elaborate on the term, they would be asked to narrate their healthcare experiences, paying specific attention to care processes involving multiple providers.

### Data processing and analysis

Interviews were recorded and subsequently transcribed by principal investigator and a co-investigator. Randomly picked samples of the transcripts were checked for accuracy against notes taken during the interviews. Themes and sub-themes were identified following data familiarization. All themes, divided by class of organization, were sorted and consolidated through an iterative process of discussions according to Valentijn’s framework of integrated care [11]. The conceptual framework combines the functions of primary care with the dimensions of integrated care at different levels- micro (clinical integration), meso (professional and organizational integration) and macro (system integration). The framework served as the basis for transcript coding and data organization. Concepts and themes were mapped based on the aspects and levels of integration described, and subsequently reported and discussed based on our study objectives. Data management and coding were performed on QSR International's NVivo Version 10.

## Results

A total of 29 face-to-face semi-structured interviews were performed, with each lasting an average of 40 minutes. The sample included five physicians, four nurses, four pharmacists, two administrators, and 14 patients and caregivers from different care settings. Five healthcare providers, and three patients and caregivers declined participation. Additionally, two FGDs with a group of eight junior allied health professionals, and a group of four caregivers were also conducted. Participants’ characteristics are summarized in Table 1.

**Table 1:** Characteristics of participants.

**Semi-structured interview**			
**Characteristic**	**Senior Providers (n = 10)**	**Junior Providers (n = 5)**	**Patients/Caregivers (n = 14)**
**Length of experience/ exposure to healthcare**, Median years (Range)	21 (17–33)	3 (2–10)	2 (1–16)
**Specialty, n**			
Medical	2	0	-
Surgical	2	0	-
Allied Health	2	2	-
Nursing	3	1	-
Management	1	2	-
Not applicable	-	-	14
**Care setting, n**			
Inpatient	-	-	6
Outpatient	-	-	8
Not applicable	10	5	-
**Focus Group Discussions**			
**Characteristic**	**Senior Providers**	**Junior Providers (1 session, n = 8)**	**Patients/Caregivers (1 session, n = 4)**
**Length of experience/ exposure to healthcare**, Median years (Range)	-	3 (1–5)	2 (1–2)
**Specialty, n**			
Allied Health	-	8	-
Not applicable	-	-	4
**Care setting, n**			
Inpatient	-	-	0
Outpatient	-	-	4
Not applicable	-	8	-

### Understanding care integration

Patients and caregivers were unfamiliar with the term. They would attempt to explain care integration literally. Some participants suggested care integration as “one-stop care” or other similar ideas, where care needs can be fulfilled at a single location:

“*Multiple layers coming together and creating a very unique environment for the patient, for recovery*.”

- (C1, 2 years exposure, Outpatient).

When asked to describe what might constitute “a unique environment” and what the “multiple layers” were, the participant was not able to explain. Other interpretations revolved around person-focused or practice-focused factors, such as timely updates on treatment or progress for patients, having infrastructure that facilitate healing, sharing of medical records among providers, having easy access to care, and developing “*after-sales service*” that addresses patients’ emotional needs after treatment delivery:

“*It’s about informing the patient how they will be taken care of*”

- (C2, 6 years exposure, Inpatient)

“… *help patients better cope not just (with) their own symptoms… but also (focuses on) the quality of life”*

- (P9, 2 years exposure, Outpatient).

Some patients were candid about their lack of knowledge and ability to navigate through the complex healthcare system. However, they were not willing to admit to such inadequacies to healthcare providers, in part due to the embarrassment associated with their ignorance:

“…*Fear and… inferiority. They do not want people to know their problems. They want to keep it private… they are not ready to (seek help)*.”

- (P4, 2 years exposure, Outpatient)

The understanding of integrated care among junior healthcare providers seemed to be service-oriented and related to work processes:

“*(Integrated care) is about integrating care across the whole continuum from acute care to community care and then subsequently as the patient recovers, it will include outpatient care*.”

- (J4, 3 years of service, Allied Health)

“… *Chronic disease services are arranged or organized around the patient*, *at the right time*, *at the appropriate time of need”*

- (J5, 2 years of service, Administrator).

Beyond definitions, most frontline staff had difficulties relating integrated care to initiatives such as the RHS. They described RHS inaccurately or incompletely. Although one participant was able to relate RHS to regional healthcare, she was not able to explain in greater detail of how exactly RHS integrates care.

“*I think regional health system sets the practice standard*, *a certain kind of standard or perspective to make sure that we are able to integrate with the rest of the world*”

- (S4, 17 years of service, Nurse)

“*RHS means it’s regional… that on top of the Ministry of Health*, *on top of the healthcare clusters we have*, *RHS oversees the whole system… I do not know specifically what they do*, *but I understand they try to connect us to the external organizations*, *whom we can (then) work with*.”

- (S5, 17 years of service, Nurse)

Among senior providers and administrators, the understanding was generally more comprehensive, covering a range of issues from system-wide resource allocation, models of care, health promotion, and disease prevention. In addition to clinical care and elements of patient-centeredness (provision of care that is respectful of, and responsive to, individual patient preferences, needs and values, and ensuring that patient values guide all clinical decisions) covered by junior providers, they discussed efficient use of resources at the organizational and systems levels [27].

“*Care that is integrated across a care continuum using the appropriate resources at the appropriate time with the maximum efficiency… That is care integration. I want to make sure the patient gets the right level, right kind of care by the right specialist, right person across the care continuum, and at a price that is efficient and cost-effective*”

- (S10, 30 years of service, Clinician & Administrator).

One senior clinician administrator described care integration in the context of population-based healthcare:

“*It means longitudinal health*, *it means primary prevention all the way to ILTC (intermediate and long-term care)*, *end-of-life*. *It means the fundamentals of wellness versus sickness*. *It means a vertical and horizontal integration of care delivery to deliver on that vision*.*”*

- (S8, 33 years of service, Clinician & Administrator).

### Key challenges in care integration

Patients, caregivers, and junior providers gave extensive responses around issues of clinical and service integration. Lack of institutional capacity and resources that led to poor care accessibility and long service wait time were repeatedly mentioned:

“*The ratio of therapists to patients is quite shocking*. *Fortunately*, *my case was quite mild*, *and I was able to do some exercises on my own*. *There were some (with) more serious (problems) who really needed to have a therapist beside (them) all the time*. *And you’d see them waiting*, *taking turns to wait for the therapist*.”

(P6, 3 years exposure, Outpatient)

The difference between private and public healthcare as a barrier to integrate care was mentioned. Participants explained the difference in terms of training, level of expertise and the different financing mechanism between public and private healthcare:

(1) Price difference between private and public healthcare:

“*Patients tend to stay within the public healthcare sector because they are heavily subsidized, and it costs more to move out to private sector, and because of that, we find that we (public healthcare providers) seem to be overloaded but at the same time, GPs and all that in the private sector tend to be under-utilized. This not only extends to funding but also to CSC (civil service medical benefits) beyond (public) institutions*.”

- (S7, 28 years of service, Allied Health)

(2) Complex government subsidy and assistance schemes for care in the community:

“*Individuals like myself who has been trying to figure out all the schemes. I think it’s (too) sophisticated (for us to utilize)* …”

- (P6, 3 years exposure, Outpatient)

(3) Perception that private healthcare is focused on profit maximization:

“*I have read about it*. *There was an article not too long ago about GP overcharging patients*. *They run their own (business)*.*”*

*- (P9*, *2 years exposure*, *Outpatient)*.

One of RHS’s objectives is to increase the capacity of primary care to play a greater role in chronic disease management. Participants seemed aware of the desired role of private GPs but have reservations about their competency and compared them unfavorably with hospital-based specialists.

“*For a while*, *I was going from one GP to another because I wasn’t sure*. *Every time I went to one and I thought I’ve settled down already*, *then they did something funny and really odd*, *and I went like (sigh)*, *competent or not*? *Then I moved (to another provider)*.”

- (S6, 20 years of service, Allied Health & Administrator)

“*They think the GPs know nothing*, *only cough and cold*… *actually they are well-trained*, *but after a while*, *because the only see patients who come to see them are (for) cough and cold*, *some doctors (will be) terrified to receive (complex) cases*.*”*

- (S1, 18 years of service, Clinician)

Senior providers primarily focused on issues at the meso and macro environments. In addressing poor care accessibility, one noted that there have been many right-siting (provision of care at the appropriate setting) projects shifting hospital-based patients to private primary care but due to inadequate oversight and consolidation of efforts, inefficiencies and wastages occurred:

“*It’s ridiculous*. *You look at the (right-siting project)- they have a highly paid*, *highly qualified person sitting there the whole morning and waiting for 1 or 2 patients*. *You look at the numbers- the entire program has (about a) thousand (patients)*. *How many patients come through our (regular outpatient) clinics*? *How could it ever scratch the surface of our long waiting time*?”

- (S8, 33 years of service, Clinician & Administrator)

On national policy, a few senior providers felt that some healthcare strategies were only reactionary to existing problems instead of being able to systematically address incoming healthcare needs and demographic changes in the country:

“*They (the ministry of health) only started to recognize the ferocity of aging population and acted on it in big ways a decade ago… building new hospitals one after another. Then after that, they realized there were not enough healthcare workers and so they built new medical schools, increased intake (for healthcare-related courses) drastically in the last 5 years. But what they failed to understand is that while infrastructures are easy to build, healthcare professionals especially doctors and specialists take a long time to train and be ready for service. So, the healthcare capacity crunch of today is not going away anytime soon*.”

*–*(S9, 30 years of service, Clinician & Administrator)

Aside from insufficiency in resources and capacity in the public healthcare sector, overemphasis of short-term performance indicators by the authorities was brought up:

“*The longer-term philosophy is not clear. I mean (of) your six (national) priorities, five of them are about decongesting the hospital. That’s not population health, there’s no care coordination, that’s just decanting (patients). If the purpose of care coordination is to shorten the hospital stay… to get the person out to home care, then we’ll just (be) prolonging the inevitable, which is the next hospital visit*.”

- (S8, 33 years of service, Clinician & Administrator)

In nurturing future healthcare professionals, some felt that the current medical education over-emphasizes specialization and understates holistic approach to care and collaboration. This creates a barrier to shared care between providers across different settings, and promotes the “territorial” practice we observe today where providers are often only willing to manage what is considered as their “area of care responsibility”:

“*Doctors are trained to respect fellow professionals’ opinion and judgment*. *So*, *if you are cardiac (specialist)*, *if I’m generalist*, *I will probably not touch the cardiac medicines*. *I’m not familiar and you’re the specialist*.”

- (S7, 28 years of service, Allied Health)

“*The (community) rehab care is pretty much standalone. They don’t feedback to (the) main (care provider)*.”

- (J5, 2 years of service, Administrator)

### Future changes required for care integration

Views on future changes required were consistent with perceived challenges. Three common themes were highlighted: greater involvement of patients; better alignment between operationalization and vision of healthcare policies at the national level; and wider adoption of volunteerism in the community.

Greater patient and caregiver involvement was mentioned to be important to individual’s ability to improve self-care:

“*…it’s (about) the individual making effort*, *(taking) initiative*, *be resourceful*. *After all*, *if you want to benefit from the system*, *you must also be willing to do things yourself*”

(P1, 3 years exposure, Outpatient)

However, such recognition among some patients does not always translate into tangible actions in reality. Nurses interviewed noted reluctance among patients and family members to do more in self-care and care of their loved ones:

“*They expect everything to be done by nurses*. *When we wanted to start (caregiver) training with family members*, *they give many excuses*.”

- (S3, 20 years of service, Nurse)

“*They see the hospital like a hotel because they (are well looked after here)*. *They can utilize their Medisave*, *and (if) they have no cash*, *Medifund is available*.”

- (J2, 10 years of service, Nurse)

When probed to suggest changes to improve patient and family involvement, only a few patients agreed that they have greater roles to play, such as engaging in lifestyle changes to improve health. Caregivers felt that their ability to care for family members with chronic disease is stifled by other commitments in life. They would rely on healthcare providers or domestic helpers for long term care needs. None of the patients and caregivers spoke about confidence in improving their own level of healthcare knowledge.

An interesting proposal from a caregiver was to intensify health education and first aid training to school children in preparation for future caregiving needs.

Among providers interviewed, some felt consumer voices should be heard while others expected more practical alignment between implementation of care integration programs with high level visions:

“*(We) always say we need to engage our patients (in infrastructural and service planning)*, *and we need to get (their input) … (but) how many committees (do) we have patients (involved)*? *Zero*. *None*. *Then*, *what do we do*? *Surrogate patients (by our own staff)*. *But you know*, *it is different versus bringing in the (real) patient*, *right*?”

- (S6, 20 years of service, Allied Health & Administrator)

“*We (should) design our physical environment*, *create opportunities for recreation better so that we can encourage people to exercise more*, *live healthier lifestyles*. *Can we also… make healthier (food) options much more visible*, *available and affordable*?”

- (J5, 2 years of service, Administrator)

A clinician administrator specifically hoped to see more development in volunteerism in the community and suggested “time banking”, where one may save time credits by volunteering and be able to “withdraw” and utilize them when needed to be cared for by volunteers in the future. Another participant suggested the need to think ahead of the current aging population and chronic disease challenges to facilitate development of advance care planning for the future:

“*People are going to die*, *people are going to get cancers*. *So*, *you have to teach people to manage and live through the end-of-life issues… talk about slow medicine and (advance care planning)*.*”*

*(S10, 30 years of service, Clinician & Administrator)*.

## Discussion

In summary, our study of stakeholders’ view of integrated care yielded heterogeneous responses and painted a complicated picture. Patients, caregivers, healthcare providers, and leaders understood the concept of care integration differently and perceived challenges for the healthcare system in the future at different levels of society. There is a mismatch with what patients and caregivers prioritize as important in receiving care versus what providers feel would be important in their work. The mismatch would have practical and policy implications.

### Changing the conversation of integrated care

Despite government’s effort to explain RHS and the goals of care integration to the public, patients were unaware of RHS and that they were receiving care in an institution within an RHS. Overwhelming majority of patients and caregivers understood integrated care in terms of costs and accessibility. They focused on individual’s care needs and expressed ideas related to clinical, functional and financial integration. For example, they spoke about long wait time and perceived lack of coordination in how they paid for healthcare. Patients didn’t speak about team-based care, patient-centeredness, and the link between social and healthcare needs–areas commonly associated with care integration. While CHAS intended to encourage utilization of private primary care, patients regarded them as nothing more than financial assistance.

The incomplete understanding of integrated care and related policies are not surprising. For patients, they are concerned with the day-to-day immediate healthcare needs and how their healthcare conditions might be a burden to their families. The situation in Singapore is compounded by the fact that older Singaporeans have little formal education. Technical terms associated with clinical care (such as care coordination) [28] would be a challenge to grasp, let alone systems-related ideas. If it is regarded that patients are active partners in the healthcare system, we would need to find better ways to explain care integration. Some innovative ways are being trialed in Singapore, including using soap opera in dialect language that elderly patients may find easier to relate to and willing to watch [29], and enlisting retired volunteers as policy ambassadors to explain government initiatives, door-to-door, in the community [30–31]. Some of these may be improvised or emulated in other countries or settings.

Junior providers’ perception of care integration reflects what it is important to them–work processes. Much of what they explained has to do with operational matters, such as coordination of processes between departments, better sharing of information among providers. Senior providers and leaders, on the other hand, have their priorities set around system integration with improved models of care, intersectoral collaboration and population-based care. These differences in views and priorities between senior leadership and junior providers need to be bridged. Healthcare leaders would benefit by listening to providers on the ground, understand how initiatives to integrate care they introduce may have translated to more work for individuals or confusion when work processes are changed to accommodate care integration.

The differences in understanding of care integration could have contributed to differences in expectations for the role of the healthcare system. While providers value care coordination and efficient use of healthcare resources in the form of right-siting, patients and caregivers do not seem to share such priorities. Patients value good service quality, convenience and ready access to public healthcare regardless of the nature of their problems. It is important to bridge such disconnect in expectations, and get providers to understand that patients are indifferent to how services are organized as long as they receive good care. To improve providers’ awareness of what patients want, creating platforms for patients’ voice to be heard would benefit everyone. Such a platform could come in different forms, including formal representation of patients in healthcare planning for RHSes; formation of patient advocacy groups, particularly for patients with chronic disease management needs; and to have public forum that would allow patients to share their views on how care integration policies have affected their care experience. In other tangible ways, public healthcare providers may actively advocate accessing appropriate level of medical care to patients, educate them whenever misinformation about the healthcare system surfaces, and strengthening their confidence towards other providers especially in the primary care sector.

### Reconcile with challenges and expectations

Participants gave a wide range of views on challenges and expectations of our healthcare system and integrated care. Among patients and caregivers, a major challenge repeatedly discussed was a perceived inadequacy in healthcare resources available to them, while providers perceived more shortcomings in national policies and lack of participation in care processes by patients and caregivers. We believe that beyond the availability of resources, possible imbalances in their allocation could be an important contributing factor affecting downstream service quality, accessibility, and public-private partnership. An England’s King’s Fund report [32] also noted such findings consistently in their studies. In Singapore, the government has been providing generous support to public healthcare institutions in terms of subsidies [33], infrastructures and human resources, while maintaining a non-interference approach to private healthcare sector [34]. While such policy was suitable in the past where care needs were episodic and care burden was not as high, it is straining our public healthcare system today as many elderly patients with chronic care needs continue to rely heavily on public healthcare [35–37]. The strain was keenly felt recently when bed crunches and service inadequacies made headlines in Singapore [38–40].

Our study findings also revealed a “specialists-know-best” mindset among patients and caregivers, and an apparent lack of confidence towards private GPs’ capabilities. The sum effect of these factors impacts the development of primary care [41] and can negatively influence the outcomes of patients with multiple comorbidities.

While confidence for and capabilities of primary care providers may take a longer time to build, improvement in existing inter-professional collaboration and sharing of information across institutions and practices could be strengthened. For instance, though a National Electronic Health Record was launched in Singapore in 2011, adoption by GPs have been slow [42] due to administrative barriers such as hardware availability and patient confidentiality issues. A survey of ten advanced countries [43] revealed that low adoption rate of healthcare informatics was also prevalent among them. The urgency of the issue was highlighted in a Commonwealth Fund’s international survey among GPs in 2012, where majority of GPs reported inadequate flow of medical records and information between hospitals and primary care, with less than half of the respondents saying they always know about changes to their patients’ medications or care plans [44]. In realizing the vision of “One Patient, One Record”, more GPs, especially the older generation, need to be encouraged to adopt and contribute to shared medical records of our patients.

There were some discussions about shared care beyond short term measures that simply decant patients from hospitals to the intermediate or community care institutions. The acute hospitals within each of the RHSes could start by moving away from hospital-centric approaches and reaching out to community-based providers, to strengthen relationships. For example, co-location of hospital-based providers in community settings [45] would extend not only specialist care but also build trust with community providers, giving patients the sense that “shared care” is indeed taking place.

Furthermore, priorities among healthcare professionals within each institution need to be aligned. While providers in the leadership position focus on high-level policies and assume downstream development of work processes would ensue smoothly, junior providers may not appreciate those priorities to similar extent and instead expect clear instructions to be given to them. To narrow the gap, senior leaders need to understand the challenges and implications, such as having to fill extra forms, learning multiple information systems, and reconcile differences in care pathways, that come along with care integration initiatives—all of which could cause confusion and would take time to streamline.

Our study participants rarely mentioned social dimension of care delivery. Very often patients with chronic diseases require more social support [46] than healthcare providers realize. The lack of emphasis to social care may be a result of extensive pre-occupation with conventional goals of therapies in medical care settings. There is indeed a need to provide enabling environment that allows patients to heal and stay healthy away from healthcare institutions. Moreover, the aged and sick are not necessarily dependents all the time. Ways to keep them active and positively connected to the wider society should be constantly explored. At the community level, it will be beneficial to forge a supportive environment through volunteerism where not just immediate family members, but members of the community also look out for one another.

Only few mentioned patient-centered care, or how care should be customized to support patient and family involvement. Such phenomenon may be due to the prevalent Asian culture where patients are more deferential towards people with power (doctors); they are also less educated about healthcare delivery; and importantly, providers often do not invite patients to participate in decision-making. Thus, in our study, while some providers shared the hope of seeing more active participation by patients in the care process, patients and caregivers interviewed seldom spoke proactively about what they could do for themselves or their loved ones at the individual or community level. Instead, patients suggested for more governmental resource commitment for infrastructural and healthcare human capital development for the future. This may be that healthcare has been experienced in a one-directional manner for the past few decades. Patients are simply passive consumers of care services. As a result, knowledge and the underlying confidence in managing one’s own health are weak. Improving knowledge and self-confidence will be an important step in enhancing patient and family involvement in chronic care. Increasingly, we observe more patient education activities being carried out by various healthcare professionals. While such activities may not yet yield significant results, we are cautiously optimistic about potential positive impacts to patient empowerment in the near future.

A lot of the frustration and challenges discussed by the healthcare providers and consumers in our study can be traced to the combined effect of rapid demographic changes, public policies over the years and inability of our patients’ health awareness to keep up with the pace of rapid healthcare development. Many of the cultural and cognitive barriers to integration discussed by our study participants have also been reported in the literature [47–52]. To mitigate barriers to alignment of understanding and perception, a new set of vocabulary may be needed for public education; To strengthen private primary care, more opportunities, such as allowing service obligation to be fulfilled outside public institutions, are needed for new doctors to enter the sector. Besides, the goals and targets set for public healthcare institutions should diversify from the current obsession with immediate tangible outcomes, such as readmission rates and associated cost savings that encourage the practice of decanting patients, to performance-based outcomes that may help promote shared care.

### Strengths and limitations

The use of semi-structured interviews enabled study investigators to prepare questions ahead of time. It followed a discussion track while allowing participants certain freedom to their views and experiences in their own terms. Such method allowed comparisons of comments to be made among interview participants for easy categorization of ideas and themes. For patients and caregivers, focus group discussions were useful in obtaining detailed information about personal and group feelings, perceptions and opinions. Views from some participants might help to elicit responses from others and the method provided a broader range of information. In sampling of participant, due consideration was exercised to ensure there was good representation of healthcare stakeholders in the sample set. Audio recording during data collection enabled retrospective verification of data.

Our study had limitations. Some findings may lack generalizability as we conducted the study in public healthcare institutions. The use of snowball sampling for healthcare professionals might also result in early data saturation. However, as the healthcare system in Singapore is relatively porous, many participants interviewed did have exposure and knowledge of healthcare at various settings. Moreover, before snowball sampling was initiated, we attempted to ensure the diversity of our study participants by purposively selecting initial participants of different backgrounds, seniority, job functions and occupational exposures to ensure that we were able to capture a wider spectrum of views. We interviewed only English and Mandarin speaking patients. The perspectives of patients who were not fluent in English or Mandarin were not represented, giving rise to potential participant selection bias. However, as English is the official working language in Singapore and Chinese forming the majority of the resident population, the study team believes that the views captured by our sample should adequately represent the majority. Data collection was terminated only upon data saturation to ensure we have adequately capture possible ideas and opinions from the participants.

## Conclusion

As the healthcare needs change from specialized, episodic models in the past to an integrated, longitudinal model for the future, stakeholders from all levels should share common understanding and priorities for integrated care. However, ordinary healthcare consumers seem to have incomplete understanding of care integration concept and experience navigation difficulty in today’s complex healthcare environment. While healthcare providers do generally recognize the need for integration of services, their understanding and priorities are not aligned. Mismatches in perceptions and expectations highlight the urgent need to change the conversation of integrated care: patients, family members, providers on the ground cannot be left behind as the healthcare system undergoes inexorable changes in the near future.
